# ICTV Virus Taxonomy Profile: *Togaviridae*

**DOI:** 10.1099/jgv.0.001072

**Published:** 2018-05-10

**Authors:** Rubing Chen, Suchetana Mukhopadhyay, Andres Merits, Bethany Bolling, Farooq Nasar, Lark L. Coffey, Ann Powers, Scott C. Weaver, ICTV Report Consortium

**Affiliations:** ^1^​ Department of Microbiology and Immunology, University of Texas Medical Branch, Galveston, TX 77555, USA; ^2^​ Department of Biology, Indiana University, Bloomington, IN 47405, USA; ^3^​ Institute of Technology, University of Tartu, Nooruse 1, 50411 Tartu, Estonia; ^4^​ Arbovirus Laboratory, Texas Department of State Health Services, 1100 West 49th Street, Austin, TX 78714, USA; ^5^​ Virology Division, United States Army Medical Research Institute of Infectious Diseases, 1425 Porter Street, Frederick, MD 21702, USA; ^6^​ Department of Pathology, Microbiology and Immunology, School of Veterinary Medicine, University of California, Davis, CA 95616, USA; ^7^​ Division of Vector-Borne Diseases, Centers for Disease Control and Prevention, Fort Collins, CO 80521, USA

**Keywords:** *Togaviridae*: taxonomy, ICTV Report, chikungunya virus, eastern equine encephalitis virus, rubella virus

## Abstract

The *Togaviridae* is a family of small, enveloped viruses with single-stranded, positive-sense RNA genomes of 10–12 kb. Within the family, the genus *Alphavirus* includes a large number of diverse species, while the genus *Rubivirus* includes the single species *Rubella virus*. Most alphaviruses are mosquito-borne and are pathogenic in their vertebrate hosts. Many are important human and veterinary pathogens (e.g. chikungunya virus and eastern equine encephalitis virus). Rubella virus is transmitted by respiratory routes among humans. This is a summary of the International Committee on Taxonomy of Viruses (ICTV) Report on the taxonomy of the *Togaviridae*, which is available at www.ictv.global/report/togaviridae.

## Virion

**Fig. 1.**
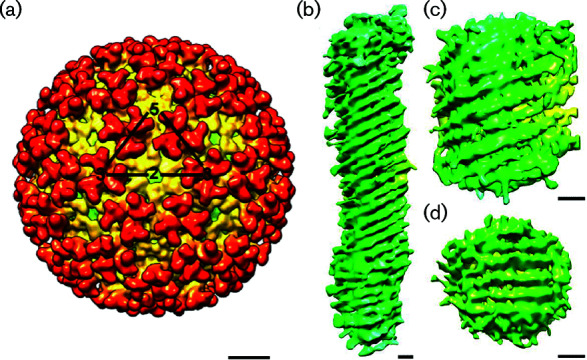
Structure of togavirus particles. (a) Three-dimensional cryo-electron reconstruction of chikungunya virus at 10.2 Å resolution (courtesy of J.  C. Y. Wang). The triangle outlines one icosahedral unit with the symmetry axes labelled. (b–d) Representation of three different rubella virions, each determined using cryo-electron tomography and no averaging procedures. The resolution of the reconstructions is not absolute, but is estimated to be better than 50 Å. All scale bars represent 10 nm. Modified from [2].

Togavirus particles consist of a nucleocapsid core, a lipid bilayer and surface glycoproteins. However, there are some striking differences between alphaviruses and rubella virions. Alphaviruses are spherical and about 70 nm in diameter. Particles contain a distinct icosahedral core and an icosahedral glycoprotein layer [1]. In contrast, rubella virus particles are pleomorphic, often tube-like and do not have icosahedral symmetry. Rubella virus capsid proteins form homodimers in a grid-like pattern and the glycoproteins are arranged in rows on the virion surface [2] (Table 1, Fig. 1).

**Table 1.** Characteristics of the family *Togaviridae*

Typical member:	Sindbis virus (J02363), species *Sindbis virus*, genus *Alphavirus*
Virion	Enveloped, 65–70 nm spherical virions for alphaviruses or 50–90 nm pleomorphic virions for rubella virus, with a single capsid protein and three or two envelope glycoproteins, respectively
Genome	10–12 kb of positive-sense, unsegmented RNA
Replication	Cytoplasmic, in vesicles derived from the plasma membrane/endosomal compartment. Assembled virions bud from plasma membrane (alphaviruses) or into the lumen of Golgi apparatus (rubella virus)
Translation	Non-structural proteins are translated from genomic RNA, and structural proteins from subgenomic RNA, both as polyprotein precursors
Host range	Humans and nonhuman primates, equids, birds, amphibians, reptiles, rodents, pigs, sea mammals, salmonids, mosquitoes and some other arthropods; most members of genus *Alphavirus* are mosquito-borne
Taxonomy	Two genera (*Alphavirus* and *Rubivirus*) including more than 30 species

## Genome

**Fig. 2.**
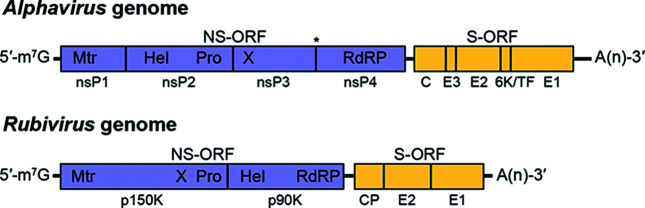
Alphavirus and rubivirus genome organisation. Black lines – non-coding regions; open boxes – ORFs (NS-ORF, non structural protein ORF; S-ORF, structural protein ORF) with processed products indicated beneath. An asterisk indicates the stop codon present in some alphaviruses that must be translationally read through to produce a precursor containing nsP4. Mtr, methyl transferase; Pro. protease; Hel, helicase; X, unknown function; RdRP, RNA-dependent RNA polymerase (courtesy of T. K. Frey).

The virus genome is unsegmented RNA of 9.7–12 kb (alphaviruses) or 9.8–10 kb (rubella virus). Non-structural and structural polyproteins are encoded by separate ORFs that are flanked by 5′- and 3′-terminal non-coding regions and separated by an internal non-coding region (Fig. 2). The subgenomic promoter overlaps with the 3′-end of the non-structural ORF and an internal non-coding region [3].

## Replication

Replication complexes (spherules) are formed on the plasma membrane. Non-structural polyproteins are cleaved in an ordered manner by a viral cysteine protease to form (1) a short-lived replication complex for negative-sense RNA synthesis and later (2) a stable replication complex for synthesis of positive-sense RNA genomes and subgenomic mRNAs. Replication enzymes also include a methyl-guanylyl transferase, an RNA helicase and an RNA-dependent RNA polymerase. The orders of these conserved functional motifs in non-structural polyproteins are different between the two genera [4].

Structural polyproteins are processed by cellular enzymes and, for alphaviruses, also by autoprotease activity of the capsid protein. The capsid protein assembles with the viral genomic RNA to form viral nucleocapsids. The glycoproteins are co-translationally inserted into the endoplasmic reticulum and translocated to the plasma membrane (alphaviruses) or to membranes of the Golgi apparatus (rubella virus). From these respective sites, alpha- and rubella viruses bud to form virions with lipid envelopes [5].

## Taxonomy

### Alphavirus

This genus includes >30 species. Most alphaviruses are mosquito-borne, transmitting alternatively between mosquito vectors and vertebrate hosts including humans, non-human primates, equids, birds, amphibians, reptiles, rodents and pigs. Chikungunya virus is an important human pathogen; most other alphaviruses cannot develop sufficient viraemia in humans to infect mosquitoes. Eilat virus [6], and probably Taï Forest alphavirus [7], are insect-specific. The aquatic alphaviruses, southern elephant seal virus and salmon pancreas disease virus, appear to be transmitted horizontally.

### Rubivirus

Members of the single species in this genus, *Rubella virus*, infect only humans and are transmitted via respiratory routes. The taxonomic relationship of *Rubivirus* to *Alphavirus* is under active assessment because of significant differences in their members’ virion structures and because their genome sequences are not monophyletic with respect to those of other positive-sense ssRNA viruses.

## Resources

Full ICTV Online (10th) Report: www.ictv.global/report/togaviridae.
